# Adsorption of Cd (II) by a novel living and non-living *Cupriavidus necator* GX_5: optimization, equilibrium and kinetic studies

**DOI:** 10.1186/s13065-023-00977-4

**Published:** 2023-06-14

**Authors:** Xingjie Li, Qiusheng Xiao, Qin Shao, Xiaopeng Li, Jiejie Kong, Liyan Liu, Zhigang Zhao, Rungen Li

**Affiliations:** 1 College of Life Science and Environmental Resources, Yichun University, Yichun, 336000 China; 2 Engineering Technology Research Center of Jiangxi Universities and Colleges for Selenium Agriculture, Yichun, 336000 China; 3 Key Laboratory of Crop Growth and Development Regulation of Jiangxi Province, Yichun, 336000 China

**Keywords:** Adsorption, Cadmium, *Cupriavidus necator* GX_5, Biosorbent

## Abstract

Biosorbents have been extensively studied for heavy metal adsorption due to their advantages of low cost and high efficiency. In the study, the living and non-living biomass of *Cupriavidus necator* GX_5 previously isolated were evaluated for their adsorption capacity and/or removal efficiency for Cd (II) through batch experiments, SEM and FT-IR investigations. The maximum removal efficiency rates for the live and dead biomass were 60.51% and 78.53%, respectively, at an optimum pH of 6, a dosage of 1 g/L and an initial Cd (II) concentration of 5 mg/L. The pseudo-second-order kinetic model was more suitable for fitting the experimental data, indicating that the rate-limiting step might be chemisorption. The Freundlich isotherm model fit better than the Langmuir isotherm model, implying that the adsorption process of both biosorbents was heterogeneous. FT-IR observation reflected that various functional groups were involved in Cd (II) adsorption: –OH, –NH, C=O, C–O and C–C groups for the living biomass and –OH, –NH, C–H, C = O, C–N and N–H groups for the dead biomass. Our results imply that non-living biosorbents have a higher capacity and stronger strength for absorbing Cd (II) than living biomass. Therefore, we suggest that dead GX_5 is a promising adsorbent and can be used in Cd (II)-contaminated environments.

**Supplementary Information:**

The online version contains supplementary material available at 10.1186/s13065-023-00977-4.

## Introduction

Heavy metals refer to a series of metals and metalloids with atomic numbers greater than 20 and elemental densities greater than 5 g/cm^3^ [1]; heavy metals may produce devastating consequences if released into the environment. Cd is one of the most hazardous heavy metals due to its high toxicity and non-degradation [2, 3]. It has adverse effects on plant growth, animal development and microbial metabolism. Furthermore, Cd accumulation poses health risks in humans through the food chain [4], leading to skeletal dysfunctions, cancer and kidney or liver damage [5–7]. Regarding this issue, effective measures for Cd pollution disposal in soil and water must be employed to protect the environment; however, this task is challenging.

Physicochemical approaches have been extensively studied and widely used in heavy metal contaminated sites due to their advantages of short remediation time and simple operation, including ion exchange, chemical precipitation, reverse osmosis and so on [8]. However, these applications are mostly ineffective and expensive, especially those that involve low metal concentrations [9]. In contrast, the use of microbiology biomass as a biosorption material for heavy metal remediation is an alternative method due to its low cost, high adsorption capacity and environmental friendliness [10–12].

Various types of microbiology-based biosorption materials, such as bacteria, fungi and algae, have been evaluated for their capacities to remove heavy metals [13–15]. Meanwhile, living and non-living bacterial cells have been extensively comparatively analysed for metal adsorption. Priya et al. [16] demonstrated that the biosorption of living biomass is higher than that of dead biomass. Zhu et al. [17] showed the same tendency, with adsorption capacities of 79.65 and 56.51 mg/g for living and non-living cells, respectively. In contrast, some researchers have indicated that non-living cells exhibit higher capacities than living cells [18, 19]. The biosorption procedure is a complex process that involves surface adsorption, ion exchange, chemisorption, complexation and so on [20]. In addition, it can be affected by adsorption factors, such as biomass amount, heavy metal concentrations, pH values, contact time and so on.

Although numerous bacterial cells have been studied for Cd (II) adsorption in the environment, developing a new high-capacity microbial biosorbent remains meaningful and challenging. *Cupriavidus necator* GX_5 (CP002878) is a Cd-resistant and Gram-negative bacterium formerly isolated from the rhizosphere soil of a local dominant plant near a Pb–Zn ore and considered as a plant-growth promoting rhizobacterium, which can assist hyperaccumulators to remediate Cd contaminated soil [21]. However, it is also considered a potential microbial biosorbent for Cd-polluted soil or water [22, 23]. The colony morphology is displayed in Additional file 1: Fig. S1. To our knowledge, equilibrium and kinetic studies of living and non-living *C. necator* GX_5 for Cd biosorption have not been investigated. Therefore, the present work aims to (1) analyse the biosorption potential of living and non-living biomass of *C. necato*r GX_5 as a biosorbent, (2) optimise the parameters involved in Cd removal efficiency, (3) evaluate kinetic and equilibrium models and (4) characterise both living and non-living biosorbent surfaces using scanning electron microscopy (SEM) and Fourier transform infrared spectroscopy (FT-IR).

## Materials and methods

### Bacterial cultivation and preparation of biosorbents

The strain of *C. necator* GX_5 was incubated in a Luri–Bertani broth medium at a pH of 7.0 ± 0.2 on a rotary shaker at 180 rpm and 28 °C until the logarithmic growth period, reaching an OD_600_ of approximately 1.0. For the living biosorbents, the biomass was collected by centrifuging at 10,000 rpm for 10 min. It was then washed three times with sterile distilled water, pre-cooled at − 80 ℃ and lyophilised 24 h with a Labconco freeze drier [24]. Thereafter, the dried pellet was ground into powder before use. For the non-living cells, the living bacterial suspension was inactivated using a high-pressure steam sterilization [25]. Dead biosorbent was prepared similarly to the abovementioned methods for the live ones. Meanwhile, the biosorbent dosages (concentrations) were calculated by grams per litre.

### Cd solutions

A 1000 mg/L stock Cd solution was made by dissolving CdCl_2_·2.5H_2_O in double distilled water. The solution was appropriately diluted to the specified concentrations according to the adsorption experiment and sterilised before use. The pH values were also adjusted by adding 1 mol/L of HCl or NaOH. All reagents used in the experiments were analytical grade and purchased from Aladdin Biochemical Technology Co., Ltd., Shanghai, China. The purity of CdCl_2_·2.5H_2_O and NaOH were 99% and 98%, respectively. The mass fraction of HCl was 36–38%, corresponding to 12 mol/L.

### Batch adsorption experiments

The adsorption capacity and/or removal efficiency of both the living and non-living biosorbents of *C. necator* GX_5 for Cd (II) were performed in batch experiments. They were conducted in 50 mL Erlenmeyer flasks containing 20 mL of Cd (II) working solution at 28 ℃ and a shaking speed of 180 rpm on a rotary shaker. The following influencing factors were evaluated: pH (3–7), biosorbent dosage (0.2–4 g/L), initial Cd (II) concentration (5–200 mg/L) and contact time (5–360 min). Other factors were kept constant (pH of 6, dosage of 1 g/L, initial Cd (II) concentration of 50 mg/L and contact time of 24 h). After adsorption, the supernatant was obtained by centrifuging the mixture at 10,000 rpm for 10 min. It was filtered through an inorganic filter membrane (0.22 μm), and Cd (II) concentration in the supernatant was assayed by graphite furnace atomic absorption spectroscopy (GF-AAS) AA800 (Perkin Elmer, USA). It was equipped with a graphite furnace, Zeeman correction, a hollow cathode lamp, and an air-acetylene burner [26]. The working current and wavelength were 4 mA and 228.8 nm, respectively. The instrument was controlled by a computer with WinLab32 software.

The adsorption capacity (*q*_*e*_) of the biosorbents was calculated using Eq. (1) [27]:

1 $$q_{e} ~\, = \,\frac{{\left( {C_{0} - C_{e} } \right) \times V}}{m}$$

where *q*_*e*_ (mg/g) defines the adsorption capacity of the biosorbent per unit; *c*_*0*_ (mg/L) refers to the initial Cd (II) concentration; *c*_*e*_ (mg/L) represents Cd (II) concentration at equilibrium or a certain time; *V* (L) denotes the volume of the working solution; and *m* (mg) represents the dry weight of living or non-living biomass.

The removal efficiency (*%removal*) was listed in Eq. (2) [27]:

2 $$~\% removal = \frac {{C_{0} - C_{e} }}{{C_{0} }}\, \times 1\,00$$

where *c*_*o*_ (mg/L) and *c*_*e*_ (mg/L) indicate the same meaning as in Eq. (1). All experiments were tested in three repetitions.

#### Biosorption kinetic studies

In the biosorption kinetic studies, the samples were collected, and the Cd (II) concentration in the supernatant was assayed at different time intervals (5, 10, 20, 30, 60, 120, 240 and 360 min). Other parameters remained unchanged (pH of 6, dosage of 1 g/L and initial Cd (II) concentration of 50 mg/L). The experimental data were fitted using pseudo-first-order and pseudo-second-order kinetic models.

The linear pseudo-first-order kinetic model was provided in Eq. (3):

3 $${\text{ln }}(q_{e} - q_{t} ) = {\text{ ln}}q_{e} ~ - \,k_{1} t$$

where *q*_*e*_ (mg/g) and *q*_*t*_ (mg/g) signify the adsorption capacity of the biosorbents at equilibrium and any given time, respectively; and *k*_*1*_ is rate constant. The value of *k*_*1*_ was obtained from the plot of ln $({q}_{e}-{q}_{t})$ versus *t* [28].

The linear pseudo-second-order kinetic model was expressed as:

4 $$\frac{t}{{q_{t} }}\, = \,\frac{1}{{q_{e}^{2} k_{2} }}\, + \,\frac{{\text{t}}}{{q_{e} }}$$

where *q*_*e*_ (mg/g) and *q*_*t*_ (mg/g) mean the same as in Eq. (3); and *k*_*2*_ implies the pseudo-second-order rate constant, which was determined by the plot of $\frac{t}{{q}_{t}}$ versus *t* [29].

### Biosorption isotherm studies

The widely used biosorption isotherms, namely, Langmuir and Freundlich isotherm models, were adopted to study the adsorption processes of living and non-living *C. necator* GX_5 biosorbents for Cd (II) at a pH of 6.0, a dosage of 1 g/L and varying initial Cd (II) concentrations (5, 10, 20, 50, 100 and 200 mg/L). The experiment was also conducted in 50 mL sterile flasks, each with 20 mL of working solution. After adsorption for 6 h, the Cd (II) concentration in the supernatant was measured based on the previously mentioned method.

The linear Langmuir isotherm model was represented as:

5 $$\frac{{C}_{e}}{{q}_{e}}= \frac{{C}_{e}}{{q}_{max}}+ \frac{1}{{{K}_{L}q}_{max}}$$

where *C*_*e*_ (mg/L) represents the equilibrium metal concentration; *qe* (mg/g) indicates the adsorption amount per unit biosorbent; *q*_*max*_ (mg/g) denotes the maximum theoretical adsorption capacity; and *K*_*L*_ is the Langmuir isotherm constant. The *K*_*L*_ and *q*_*max*_ values were calculated from the slope and intercept of the linear plot of 1/*q*_*e*_ versus 1/*C*_*e*_ [30]. To determine the favourability of the adsorption process, we calculated the dimensionless separation factor *R*_*L*_ using Eq. (6) [31]:

6 $$R_{L} = {\text{ 1}}/\left( {{\text{1 }} + K_{L} C_{0} } \right)$$

where *R*_*L*_ > 1 indicates the adsorption process unfavourable; *R*_*L*_ =1 indicates linear; 0 < *R*_*L*_ < 1 indicates favourable; and *R*_*L*_ = 0 indicates irreversible [32].

The linear Freundlich isotherm model was written as:

7 $${\text{log}}~q_{e} \, = \,{\text{log}}K_{F} \, + \,\frac{1}{n}{\text{log}}C_{e}$$

where *C*_*e*_ (mg/L) and *q*_*e*_ (mg/g) have the same meaning as in Eq. (5); *K*_*F*_ and *n* are the Freundlich isotherm constants, which were determined by the slope and intercept of the linear plot of log*q*_*e*_ versus log*C*_*e*_ [33].

#### SEM and FT-IR characterisation of the biosorbents

The surface morphological characteristic changes of both living and non-living biosorbents loaded with and without 100 mg/L of Cd (II) were determined by SEM (Sirion 200, USA). The strain suspension was prepared, washed and fixed with 2.5% glutaraldehyde at 4 ℃ overnight. The suspension was then smeared on a coverslip of appropriate size, air dried, dehydrated with ethanol of gradient concentrations (30%, 50%, 70%, 90% and 100%) and sputter coated with gold before observation [34].

The main functional groups before and after 100 mg/L of Cd (II) adsorption were analysed by a (FT-IR, Nicolet 6700, USA). The dried biosorbent was mixed with KBr (1:100), thoroughly ground in an agate mortar, pressed to transparent discs and immediately evaluated in the range of 4000–400 cm^−1^ at a resolution of 4 cm^−1^ [35].

### Statistical analysis

Data analyses were conducted using SAS 8.1. All plots were constructed using Sigmaplot 12.5. The data were evaluated using ANOVA and the least significant difference test, with a significance set at *p *< 0.05.

## Results and discussion

### Effect of adsorption parameters on biosorption

**Fig. 1:**
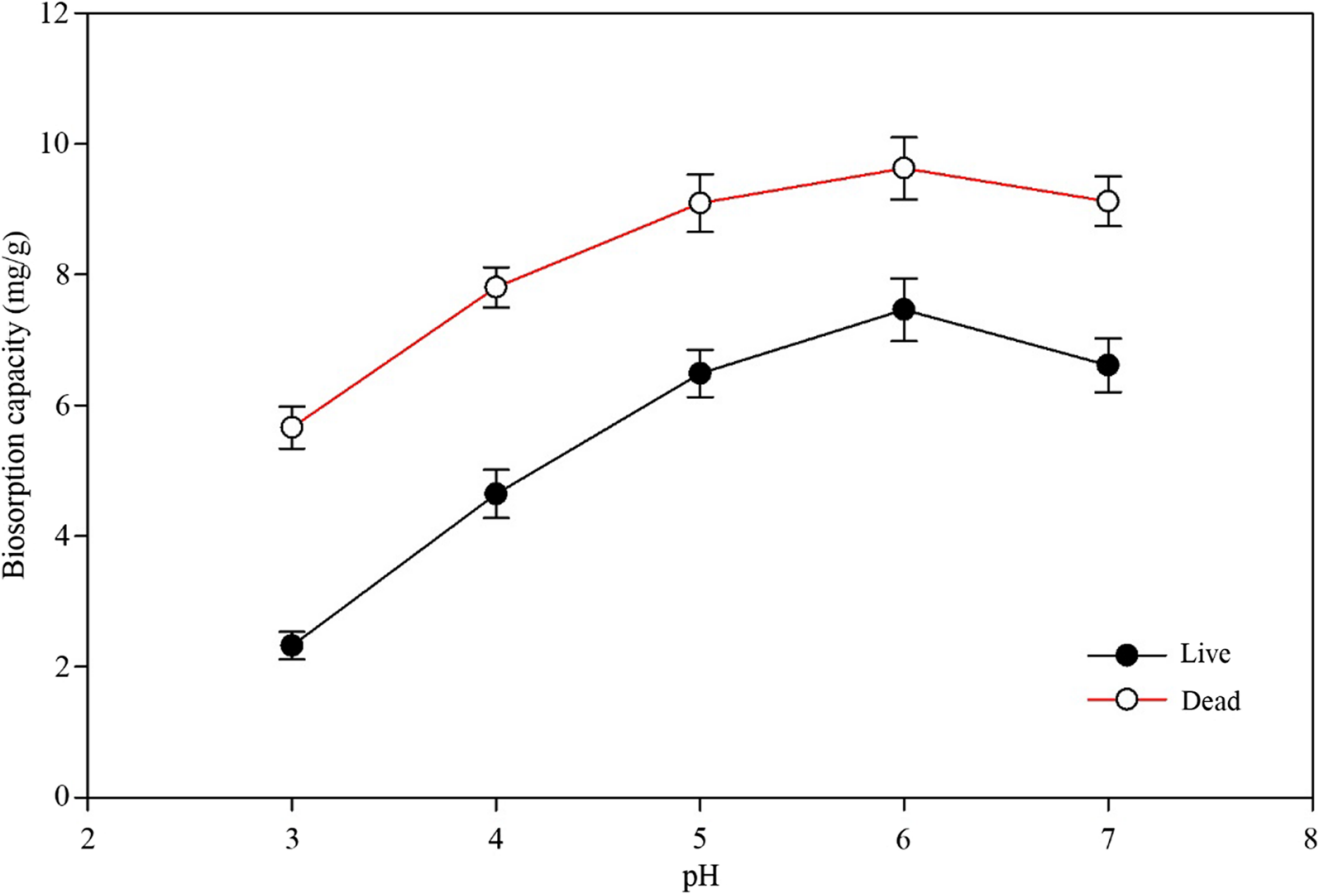
Effect of pH on the adsorption capacity of *Cupriavidus necator* GX_5

The effects of solution pH in the range of 3 to 7 on Cd (II) biosorption by the living and non-living biosorbents were studied at an initial Cd (II) concentration of 50 mg/L, dosage of 1 g/L and reaction time of 24 h. As shown in Fig. 1; Table 1, when the pH was increased from 3 to 6, the biosorption capacity of both biosorbents significantly increased and then significantly decreased for living cells at 7. Therefore, the optimal pH was considered as 6. This result may be due to the decreased competition between Cd (II) and hydroxonium ions in the solution [36]. These results agree with many other Cd (II) adsorption studies using bacterial adsorbents, which have considered 6 as the optimum pH [37, 38]. However, some researches mentioned that the maximum adsorption of Cd (II) occurs at a pH of 5 [39, 40]. In general, a pH in the range of 3 to 6 is favourable for metal adsorption by microbial adsorbents [41]. Besides, the adsorption capacity of the dead biomass was significantly higher than that of the live biomass (Table 1).

**Table 1:** The effect of pH on the adsorption capacity of *Cupriavidus necator* GX_5

Biosorbents	Adsorption capacity (mg/g)				
	pH				
	3.0	4.0	5.0	6.0	7.0
Live	2.32^f^±0.21	4.65^e^±0.37	6.49^c^±0.36	7.46^b^±0.48	6.62^c^±0.41
Dead	5.66^d^±0.32	7.81^b^±0.31	9.10^a^±0.44	9.63^a^±0.47	9.12^a^±0.38

Note: Values are the means of three replications ± standard deviation;

Means with the same superscript letter are not significantly different (p < 0.05)

Considering that hydroxyl ions react with Cd (II) and form hydroxide sediment if the pH is higher than 7, only pH values from 3 to 7 were utilised in the study. Meanwhile, in other experiments, the maximum adsorption capacity or highest removal efficiency of the biosorbents was obtained when the pH of the solution was fixed at 6. Solution pH is a vital factor affecting biosorption because it influences the surface charges and functional groups on the active sites of the biosorbents [42].

**Fig. 2:**
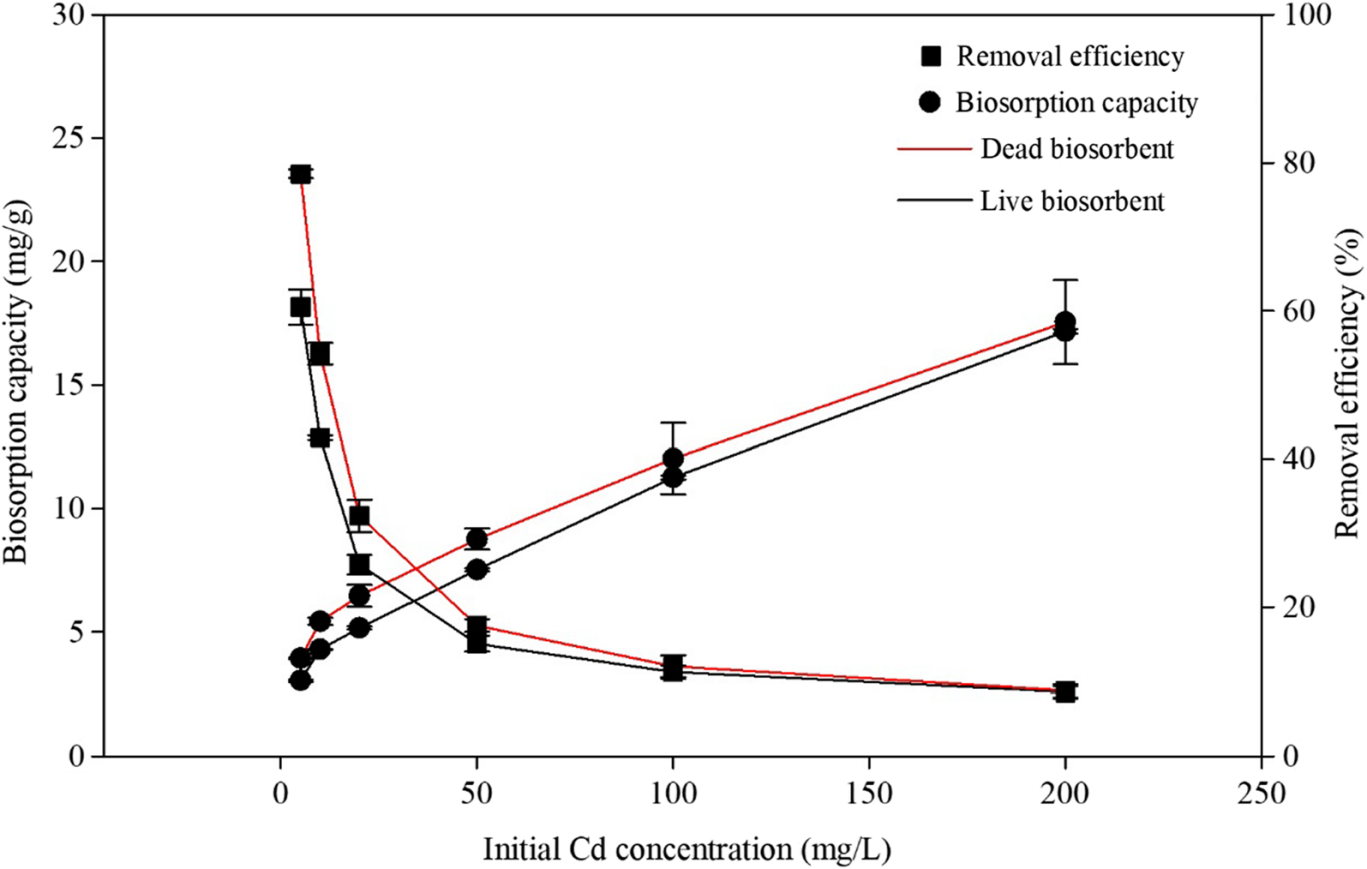
Effect of initial Cd (II) concentration on the adsorption capacity and removal efficiency of *Cupriavidus necator* GX_5

Initial metal concentration and biosorbent dosage were the other two major factors influencing the adsorption capacity and removal efficiency of the biosorbents. As shown in Fig. 2, Additional file 1: Tables S1, S2, when the initial Cd (II) concentration was increased from 5 mg/L to 200 mg/L, the adsorption capacity of the living and non-living biomass increased from 3.03 mg/g to 17.17 mg/g and from 3.93 mg/g to 17.55 mg/g, respectively. However, for the adsorption capacity of the live biomass, there existed no difference between initial metal concentration of 5 and 10 mg/L, and between 10 and 20 mg/L, while there was a significant difference between each other when the concentration was 50, 100 and 200 mg/L. For the dead biomass, the adsorption capacity was significantly different between each two concentrations from 5 to 200 mg/L, except between 10 and 20 mg/L (Additional file 1: Table S1). The biosorption capacity of dead cells seemed higher than that of the living cells (Fig. 2), but there was no significant difference (Additional file 1: Table S1).

Moreover, the removal efficiency of living and non-living biomass decreased from 60.51 to 8.58% and from 78.53 to 8.77%, respectively, and existed significant difference between each two Cd (II) concentration. Furthermore, the removal efficiency of the dead biosorbent was significantly higher than that of the live biosorbent when metal concentration was 5, 10, 20 and 50 mg/L, but with no difference at 100 and 200 mg/L, as shown in Additional file 1: Table S2. At a low concentration, sufficient binding sites were observed on the biosorbent surface, assuring that a thorough combination of Cd (II) and the biomass occurred, leading to high removal efficiency [43]. However, the ratio of metal numbers versus the available sites increases with metal concentration; therefore, the binding sites can be completely utilised, which results in improved adsorption capability [44]. Besides, the slope of the removal efficiency curve for both living and non-living biosorbents of *C. necator* GX_5 decreased at 50 mg/L of Cd (II). Therefore, this concentration was used in other batch experiments.

**Fig. 3:**
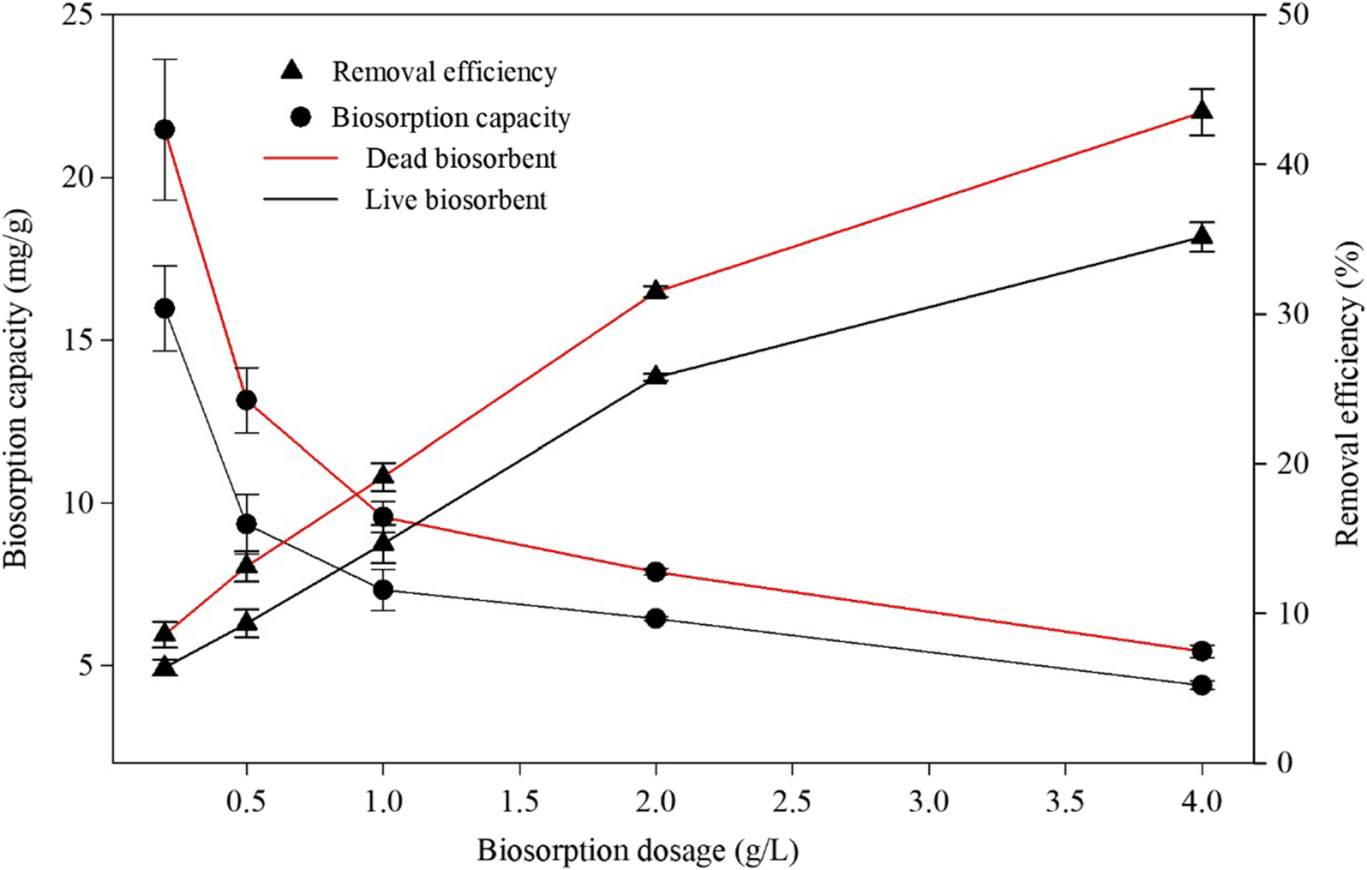
Effect of dosage on the adsorption capacity and removal efficiency of *Cupriavidus necator* GX_5

The effect of the dosage of living and non-living biosorbents on Cd (II) adsorption was investigated at an initial metal concentration of 50 mg/L, a pH of 6 and a reaction time of 24 h. As demonstrated in Fig. 3 and Additional file 1: Table S3, a decrease in adsorption capacity from 15.97 mg/g to 4.39 mg/g for the living biomass and from 21.47 mg/g to 5.44 mg/g for the non-living biomass occurred with an increased dosage from 0.2 g/L to 4 g/L. The adsorption capacity of the dead cells for Cd (II) was significantly different between each two dosages in the range of 0.2 to 4 g/L. But it was more complex for the living cells. And the dead pellet had a significantly higher adsorption capacity than live pellet at lower dosage of 1.2 to 1 g/L (Additional file 1: Table S3).

Conversely, the removal efficiency increased from 6.39 to 35.15% and from 8.59 to 43.49% for the living and non-living biosorbents, respectively. Unlikely to the presentation of the adsorption capacity, the removal efficiency between any two dosages of both live and dead biosorbents was significantly different as demonstrated in Additional file 1: Table S4. Similarly, the removal efficiency of Cd (II) by the dead pellet was significantly higher than live pellet. The increase in biosorbent dosage may aggregate or overlap, which means reducing the available binding sites, leading to decreased adsorption capacity [45]. The active sites on the cell surface cannot be fully applied, which has also decreased the adsorption capacity of the biosorbents [46]. However, the total site quantities in higher dosages were much greater than those in lower biomass concentrations, which can adsorb more metal ions, thus increasing removal efficiency [47]. Many other biosorbents for metal adsorption have displayed the same tendency [48–50]. Similarly, the slope of the biosorption capacity curve for both biosorbents decreased at 1 g/L. Therefore, the optimum dosage was 1 g/L, which was exploited in the experiments.

**Fig. 4:**
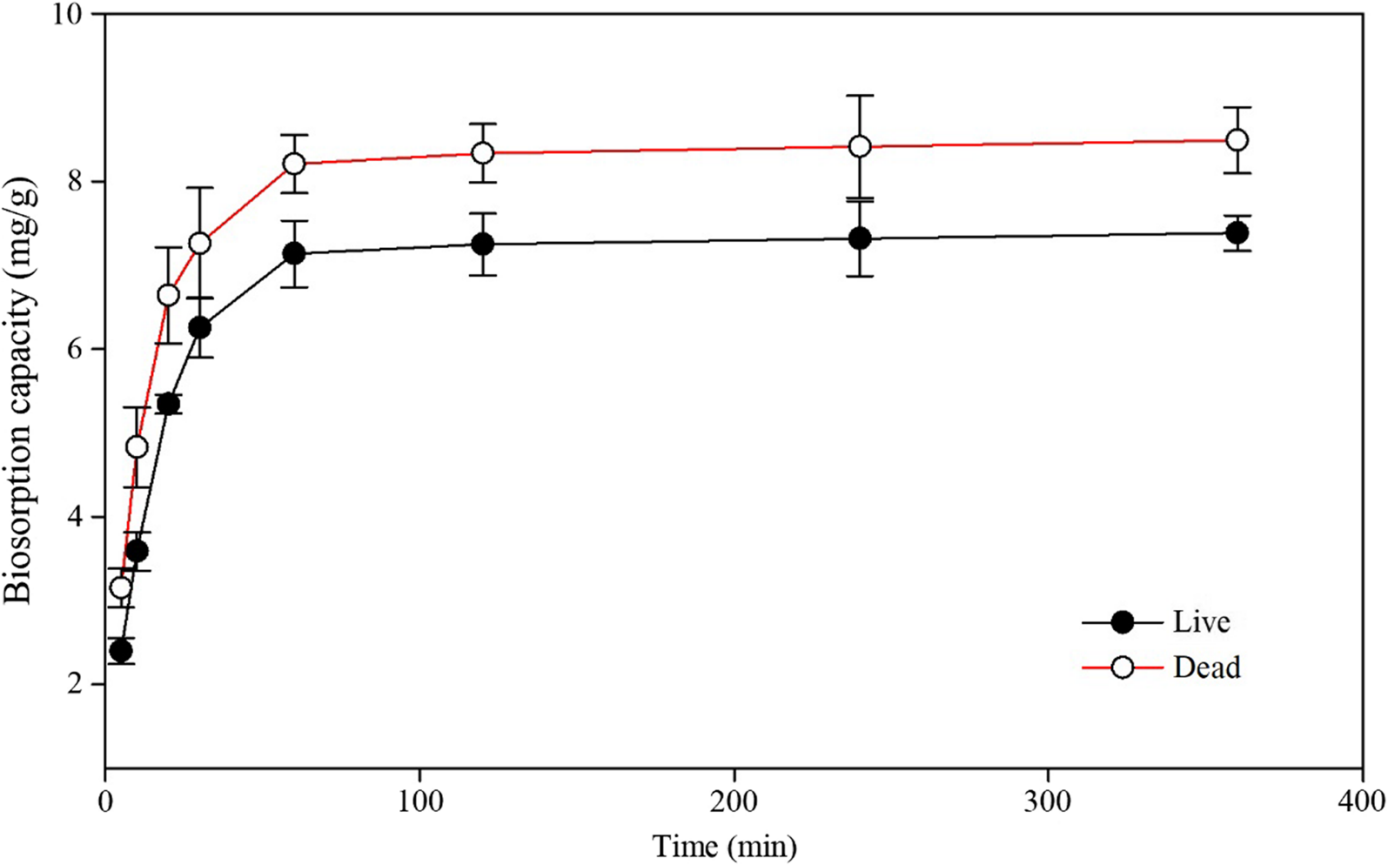
Effect of contact time on the adsorption capacity of *Cupriavidus necator* GX_5

Contact time was another important factor affecting the adsorption process. It determines the equilibrium time. The effects of contact time varying from 5 to 360 min on the biosorption capacity of the two biosorbents for Cd (II) were analysed, and the results are demonstrated in Fig. 4; Table 2. The adsorption capacity increased sharply in the first 30 min and reached adsorption equilibrium within 60 min. The rapid change in adsorption capacity in 30 min may be attributed to the many free available sites on the surface of the biosorbents [51]. In the adsorption process, the site numbers become finite, and the presence of competition of metal ions decreases the adsorption rate until equilibrium [52].

**Table 2:** Effect of the contact time on the adsorption capacity of *Cupriavidus necator* GX_5

Biosorbents	Adsorption capacity (mg/g)							
	Time (min)							
	5	10	20	30	60	120	240	360
Live	2.40^g^±0.16	3.59^f^±0.23	5.34^e^±0.11	6.25^d^±0.36	7.14^bc^±0.40	7.25^bc^±0.37	7.32^b^±0.45	7.38^b^±0.21
Dead	3.15^f^±0.23	4.83^e^±0.48	6.64^cd^±0.57	7.26^bc^±0.66	8.21^a^±0.35	8.34^a^±0.35	8.41^a^±0.61	8.49^a^±0.39

Note: Values are the means of three replications ± standard deviation;

Means with the same superscript letter are not significantly different (p < 0.05)

The rate of adsorption capacity of the living (6.35 mg/g) and non-living biomass (7.26 mg/g) at 30 min occupied 86.00% and 86.84% and 7.27 mg/g and 8.36 mg/g towards the equilibrium adsorption capacity, respectively. Other researchers have reported similar results [53, 54]. Just as discussing the effects of pH, dosage and initial metal concentration on the adsorption capacity, the dead biomass was significantly higher than live biomass at any time point of the same sampling time, as displayed in Table 2.

### Biosorption kinetic evaluation

**Fig. 5:**
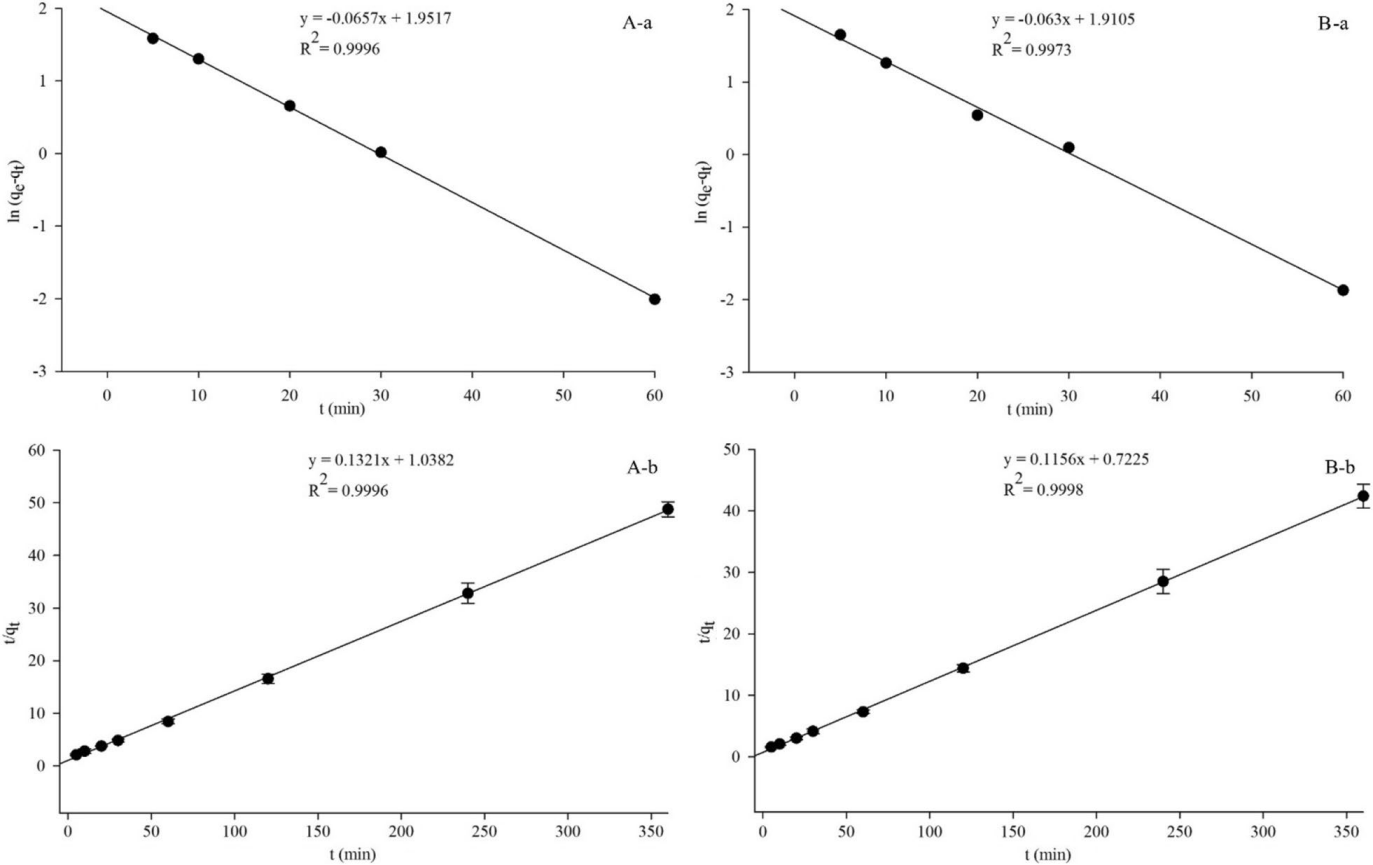
Pseudo-first-order (**a**) and pseudo-second-order (**b**) kinetic plots of the living (**A**) and non-living (**B**) biomass of *Cupriavidus necator* GX_5

To elucidate the Cd (II) adsorption process using the living and non-living *C. necator* GX_5 biomass, we tested the experimental data using the pseudo-first-order and pseudo-second-order kinetic models, which have been extensively used in metal adsorption experiments by biosorbents [55–58]. In the linear plot of ln $({q}_{e}-{q}_{t})$ versus *t*, with the reaction time proceeding, ${q}_{t}$ is infinitely close to ${q}_{e}$. Therefore, this equation is applicable only to the process before adsorption equilibrium [59]. Thus, only the experimental data before 60 min were employed to fit the parameters in the equation. Figure 5; Table 3 show that the R^2^ values of the pseudo-first-order and pseudo-second-order models were all above 0.99 for both biosorbents. However, the predicted *q*_*e*_ was lower than the *q*_*max*_ of the experimental data in the pseudo-first-order model, especially for the non-living biomass, at 6.76 mg/g in comparison to 8.36 mg/g.

**Table 3:** Adsorption kinetic parameters of Cd (II) by live and dead biomass of *Cupriavidus necator* GX_5

Strain type	*q*_max_(mg/g)	Pseudo-first-order			Pseudo-second-order		
		*q*_e_(mg/g)	*k*_1_	*R*^2^	*q*(mg/g)	*k*_2_	*R*^2^
Live	7.27	7.04	0.0657	0.9967	7.57	0.0168	0.9996
Dead	8.36	6.76	0.0630	0.9973	8.65	0.0185	0.9998

Febrianto et al. indicated that the adsorption process cannot fit the pseudo-first-order model if a large discrepancy occurs between the predicted *q*_*e*_ and the experimental *q*_*max*_ even if the plot had a high coefficient [60]. This result may be caused by the boundary layer or external resistance controlling at the beginning of the adsorption reaction, which was named as a time lag [61].

The predicted *q*_*e*_ (8.65 mg/g) in the pseudo-second order was close to *q*_*max*_ (6.76 mg/g) (Table 3.), which indicated that the pseudo-second-order kinetic model was a better fit in describing the adsorption process. This tendency suggests that the rate-limiting step may be chemisorption, complexation, coordination and/or chelation [62, 63].

### Biosorption isotherm evaluation

Two widely used isotherm models, the Langmuir and Freundlich isotherm models, were applied for the analysis of the fit of data, which can determine the adsorption affinity and capacity of the biosorbents [64]. The Langmuir model is usually used for monolayer adsorption of specific homogenous sites, whereas the Freudlich model is highly suitable for heterogeneous adsorption types of different active sites [65].

**Fig. 6:**
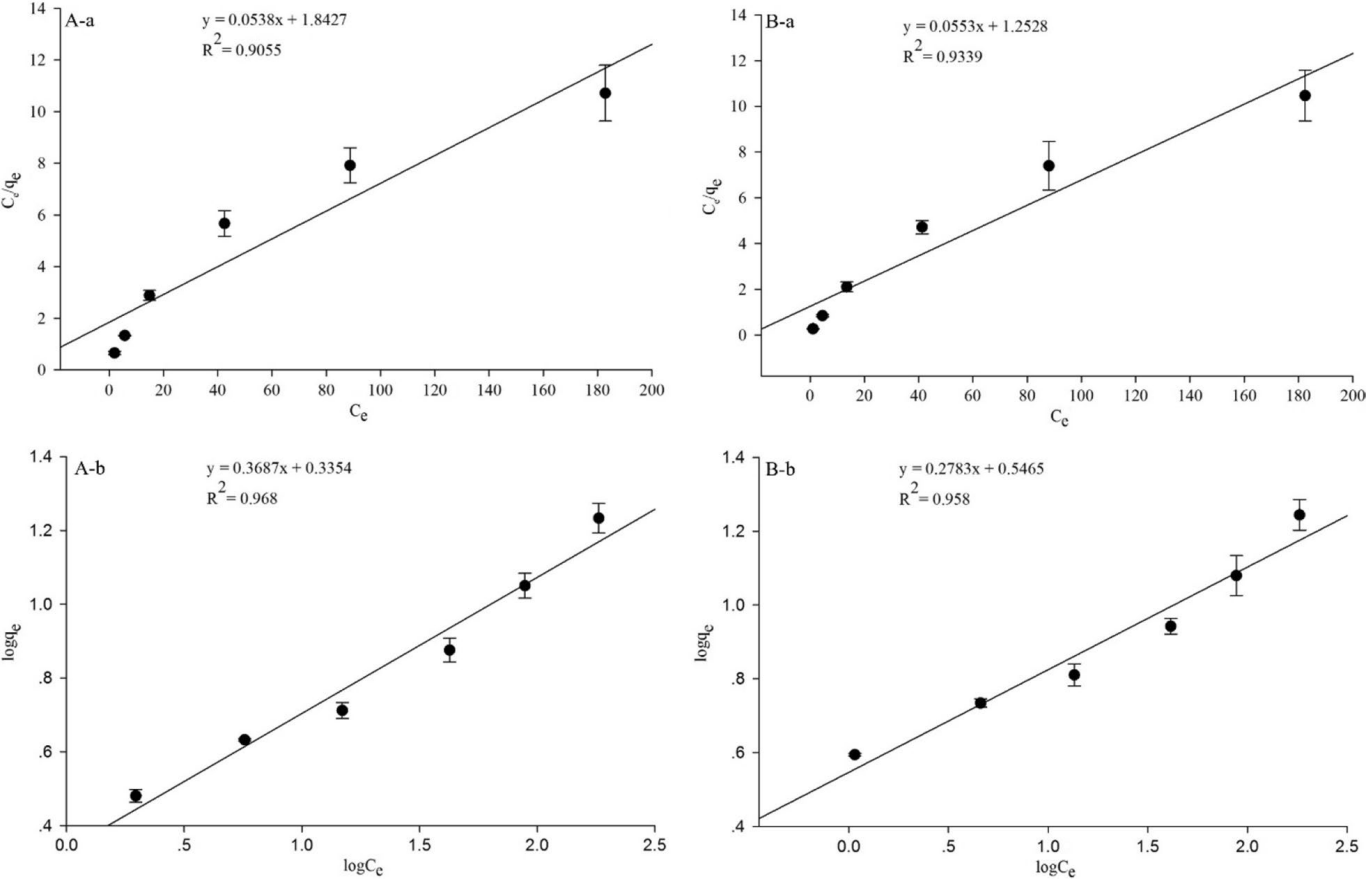
Langmuir (**a**) and Freundlich (**b**) isotherm plots of the living (**A**) and non-living (**B**) biomass of *Cupriavidus necator* GX_5

The linear plots and model parameters are shown in Fig. 6; Table 4. We determined that the R^2^ values in the Freundlich isotherm equation for Cd (II) adsorption by both living and non-living biosorbents were higher than those in the Langmuir model, indicating that the Freundlich isotherm model provided a better fit than the Langmuir isotherm model. This implied that the adsorption process of both biosorbents was heterogeneous and had multilayer adsorption.

**Table 4:** Langmuir and Freundlich biosorption constants of Cd (II) by live and dead biomass of *Cupriavidus necator* GX_5

Strain type	Langmuir isotherm				Freundlich isotherm		
	*K*_L_	*q*_max_(mg/g)	*R*_L_	*R*^2^	*n*	*K*_F_	*R*^2^
Live	0.0292	18.59	0.1462	0.9055	2.7122	2.1647	0.9680
Dead	0.0441	18.08	0.1017	0.9339	3.5932	3.5197	0.9580

The characteristics of the Freundlich equation are determined by the constants *K*_*F*_ and *n*. *K*_*F*_ is usually used to indicate the adsorption capacity of biosorbent to adsorbate; *n* means the adsorption strength, and the larger the *n* is, the stronger the reaction between the biosorbent and the adsorbate [66]. We discovered that the *K*_*F*_ value (3.5197) of non-living cells was larger than that (2.1647) of living cells in the Freundlich model (Table 4), indicating that the dead biomass had a higher Cd (II) adsorption capacity than the living biomass, which is also shown in Figs. 1 and 4; Table 3. This phenomenon agrees with experiments conducted by other investigators [67]. Meanwhile, the adsorption strength of the non-living biosorbent was stronger than that of the living biosorbent by comparing the *n* values of 3.5932 against 2.7122 from Table 4.

### SEM and FT-IR analysis

In our previous investigation, the minimal inhibitory concentration of *C. necator* GX_5 for Cd (II) was 6 mM [21]. Furthermore, the higher the metal concentration, the more evident the results would be to some extent; thus, 100 mg/L of Cd (II) was used in the SEM and FT-IR characterisation of the adsorption process.

**Fig. 7:**
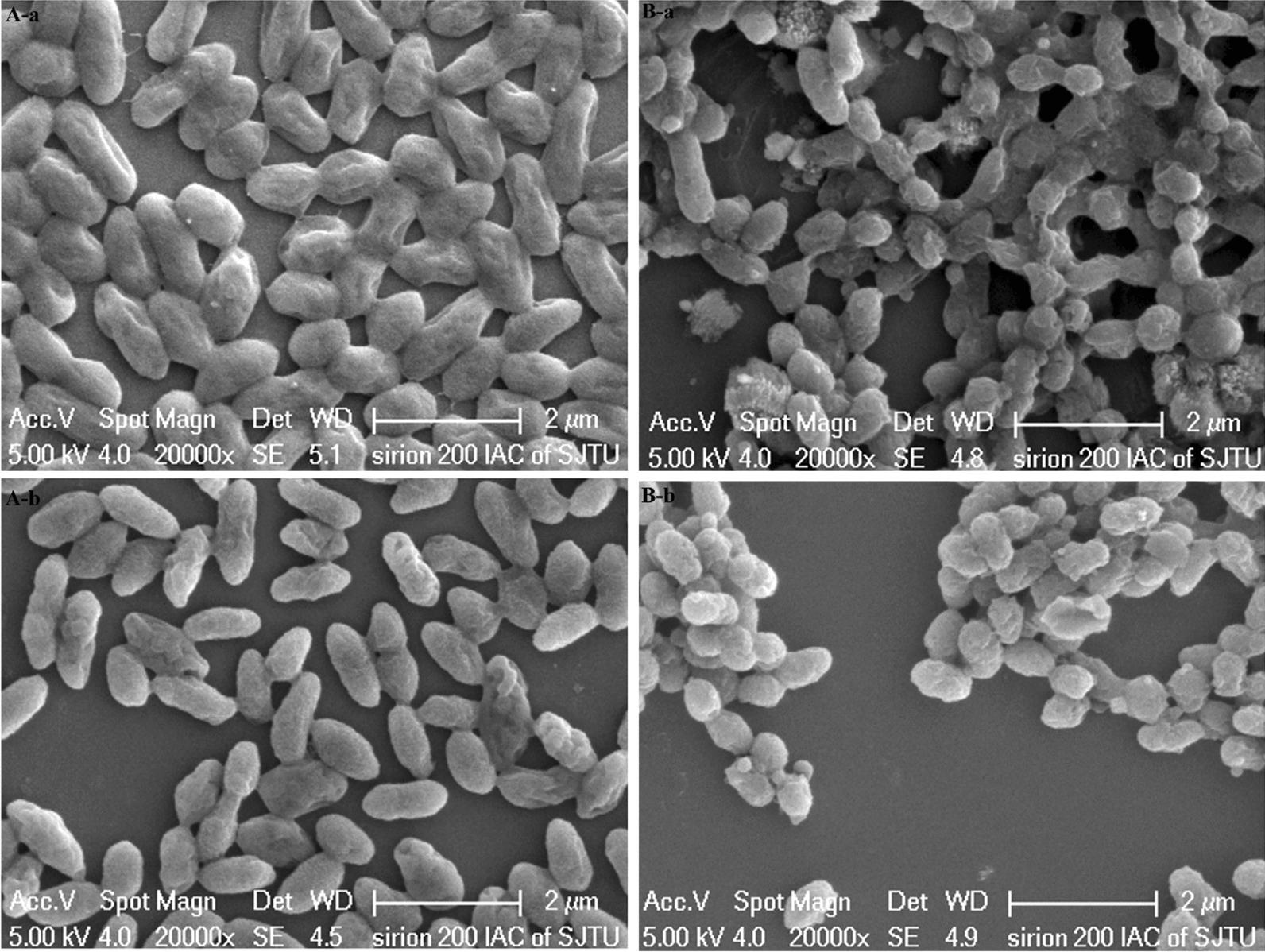
SEM images of the living (**A**) and non-living (**B**) biomass of *Cupriavidus necator* GX_5 before (**a**) and after (**b**) adsorption

The surface morphology characterisation of the living and non-living biosorbents of *C. necator* GX_5 before and after the adsorption of Cd (II) was studied using SEM, and the results are shown in Fig. 7. The surfaces of the living and non-living cells were smooth and invaginated before adsorption. In addition, the dead biomass seemed to clump. After loading with Cd (II), the invaginated parts were filled with particles and plumped, and the surface was coated with sediments. This phenomenon may be caused by the interaction of Cd (II) with microbial extracellular polymeric substances [68].

**Fig. 8:**
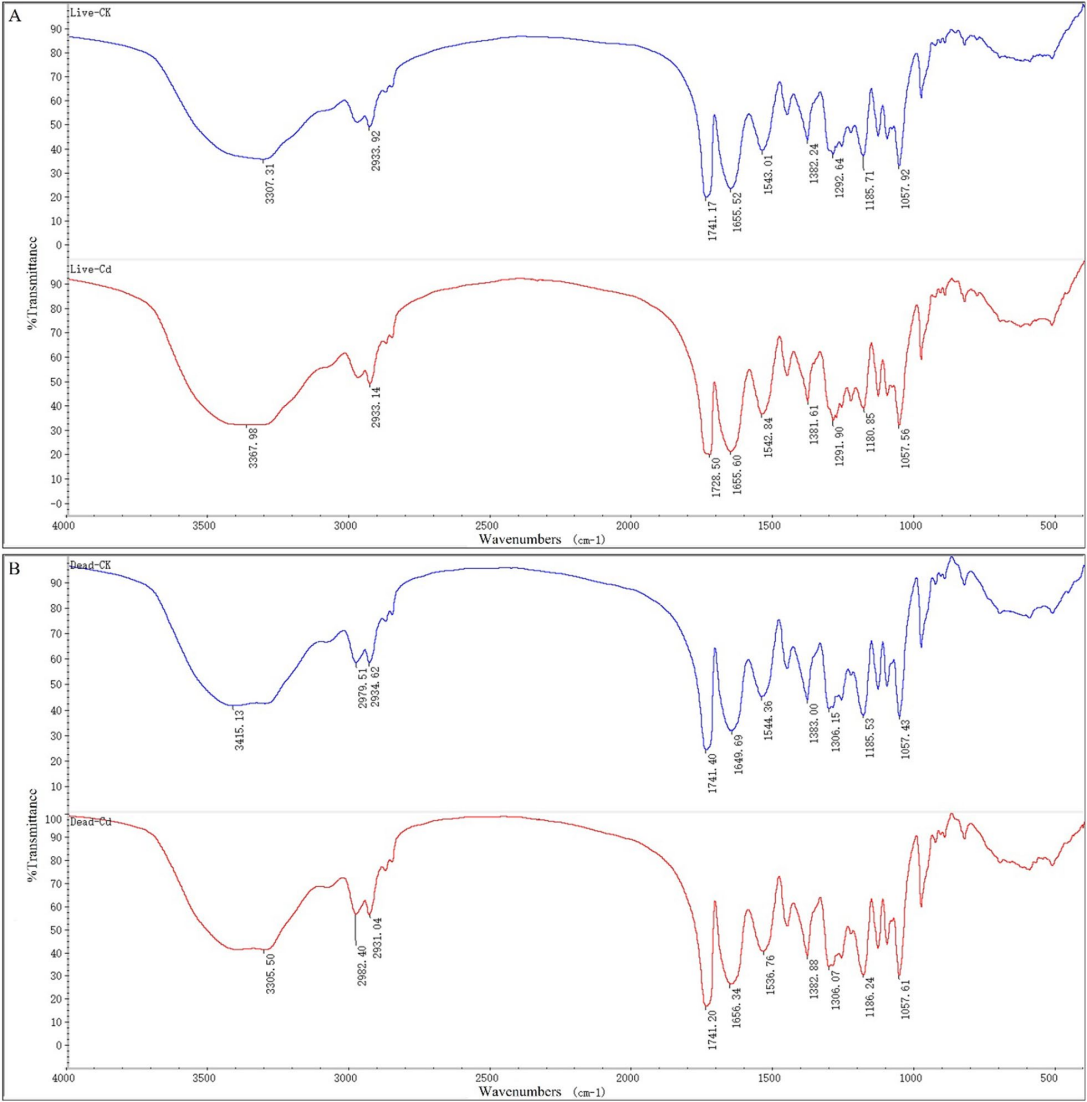
FT-IR spectra of living (**A**) and non-living (**B**) biomass of *Cupriavidus necator* GX_5 before and after adsorption

The functional groups on the cell surface were an important factor for absorbing metal ions. Changes in adsorption peaks indicate that the functional groups on the cell surface may have participated in the metal combination [69]. Therefore, the changes in the functional groups before and after adsorption with Cd (II) by the two types of biosorbents were surveyed using an FT-IR instrument. The infrared spectrum showed several different adsorption peaks, as displayed in Fig. 8. Before adsorption, the characteristic peaks of the living and non-living biomass were similar, except that a new peak at 2979.51 cm^−1^ appeared for the non-living biosorbent, which was obviously very acute. Although the peaks were similar between the living and non-living biosorbents, the action mode performed differently after Cd (II) adsorption, as demonstrated in Table 5. Xu et al. [11] studied the characterisation of Cd (II) biosorption of the living and non-living biomass of *Pseudomonas* sp. 375, and their results are consistent with ours.

**Table 5:** Main functional groups of Fourier Transform Infrared (FTIR) spectra before and after adsorption

Strain type	Before adsorption (cm^−1^)	After adsorption (cm^−1^)	Differences in shifts (cm^−1^)	Assignment
Living	3307.31	3367.98	60.67	stretching vibration of O–H and N–H of saccharide
	1741.17	1728.50	− 12.67	C=O stretching vibrations from lipids
	1185.71	1180.85	− 4.86	C–O and C–C stretching vibrations
Non-living	3415.13	3305.50	− 109.63	stretching vibration of O–H and N–H of saccharide
	2979.51	2982.40	2.89	C–H stretching vibrations
	2934.62	2931.04	− 3.58	asymmetric C–H stretching vibration
	1649.69	1656.34	6.65	stretching vibration of C= O(amide I)
	1544.36	1536.76	− 7.6	stretching vibration of C–N and deformation vibration of N–H (amide II)

For the living biosorbent, the major spectrum bands shifted from 3307.31, 1741.17 and 1185.71 cm^−1^ before adsorption to 3367.98, 1728.50 and 1180.85 cm^−1^ after adsorption, representing the stretching vibrations of O–H and N–H of saccharides [70], stretching vibrations of C = O of lipids, and stretching vibrations of C–O and C–C, respectively [71] (Fig. 8A; Table 5). Regarding the non-living biomass loaded with and without Cd(II), the main offsets were from 3415.13, 2979.51, 2934.62, 1649.69 and 1544.36 cm^−1^ to 3305.50, 2982.40, 2931.04, 1656.34 and 1536.76 cm^−1^, corresponding to the stretching vibrations of O–H and N–H [70], stretching vibrations of C–H [72], stretching vibrations of asymmetric C–H [73], stretching vibrations of C = O (amide I) [74], and stretching vibrations of C–N and deformation vibrations of N–H (amide II) [75], respectively (Fig. 8B; Table 5). All the above indicated that –OH, –NH, C =O, C–O and C–C groups might be involved in Cd (II) adsorption for living biosorbents, and –OH, –NH, C–H, C = O, C–N and N–H groups were associated with non-living biosorbents. Meanwhile, we determined that more functional groups participated in Cd (II) adsorption, which might partly explain why the adsorption capacity of non-living cells was higher than that of living cells.

## Conclusions

In this study, the living and non-living biomass of *C. necator* GX_5, which is a previously isolated Cd-resistant strain, were evaluated for their adsorption capacity and/or removal efficiency of Cd (II). The pH, initial metal concentration, dosage and contact time significantly affected the reaction process. The maximum removal efficiency rates for the living and non-living biomass were 60.51% and 78.53%, respectively, at an optimum pH of 6, a dosage of 1 g/L and an initial Cd (II) concentration of 5 mg/L. The pseudo-second-order kinetic model was a better fit in describing the adsorption process. The Freundlich isotherm model provided a better fit than the Langmuir model for both the living and non-living biosorbents. SEM analysis verified the Cd (II) absorption on the cell surface. FT-IR observation suggested that the functional groups of –OH, –NH, C =O, C–O and C–C of the living biomass and the –OH, –NH, C–H, C=O, C–N and N–H groups of the non-living biomass might be responsible for Cd (II) adsorption. This work implied that non-living biosorbents were superior to living biosorbents in Cd (II)-adsorbing capacity and strength, which is a promising adsorbent in Cd (II)-contaminated environments.

## Supplementary Information

**Additional file 1:** **Figure S1.** *Cupriavidus necator* GX_5 colonies grown on LB agar. **Table S1.** The effect of initial Cd concentration on adsorption capacity of *Cupriavidus necator* GX_5. **Table S2.** The effect of initial Cd concentration on removal efficiency of *Cupriavidus necator* GX_5. **Table S3.** The effect of biosorbent dosage on adsorption capacity of *Cupriavidus necator* GX_5. **Table S4.** The effect of biosorbent dosage on removal efficiency of *Cupriavidus necator* GX_5.

## Data Availability

All data generated or analysed during this study are included in this published article [and its supplementary information files]. Raw data can be shared via correspondence upon reasonable request.
