# Supplementary material for: Adsorption of Cd (II) by a novel living and non-living Cupriavidus necator GX_5: optimization, equilibrium and kinetic studies
Source: BMC Chem. 2023 Jun 14;17(1):54. doi: 10.1186/s13065-023-00977-4 (PMC10265800; doi:10.1186/s13065-023-00977-4)
Supplement: Supplementary file 1 — Additional file 1: Figure S1. Cupriavidus necator GX_5 colonies grown on LB agar. Table S1. The effect of initial Cd concentration on adsorption capacity of Cupriavidus necator GX_5. Table S2. The effect of initial Cd concentration on removal efficiency of Cupriavidus necator GX_5. Table S3. The effect of biosorbent dosage on adsorption capacity of Cupriavidus necator GX_5. Table S4. The effect of biosorbent dosage on removal efficiency of Cupriavidus necator GX_5. [file 13065_2023_977_MOESM1_ESM.docx]

**Supplementary Figure Legend**

Fig. S1 *Cupriavidus necator* GX_5 colonies grown on LB agar

**Supplementary Table Heading**

Table S1 The effect of initial Cd (II) concentration on adsorption capacity of *Cupriavidus necator* GX_5

Table S2 The effect of initial Cd (II) concentration on removal efficiency of *Cupriavidus necator* GX_5

Table S3 The effect of biosorbent dosage on adsorption capacity of *Cupriavidus necator* GX_5

Table S4 The effect of biosorbent dosage on removal efficiency of *Cupriavidus necator* GX_5

**Supplementary Figure**


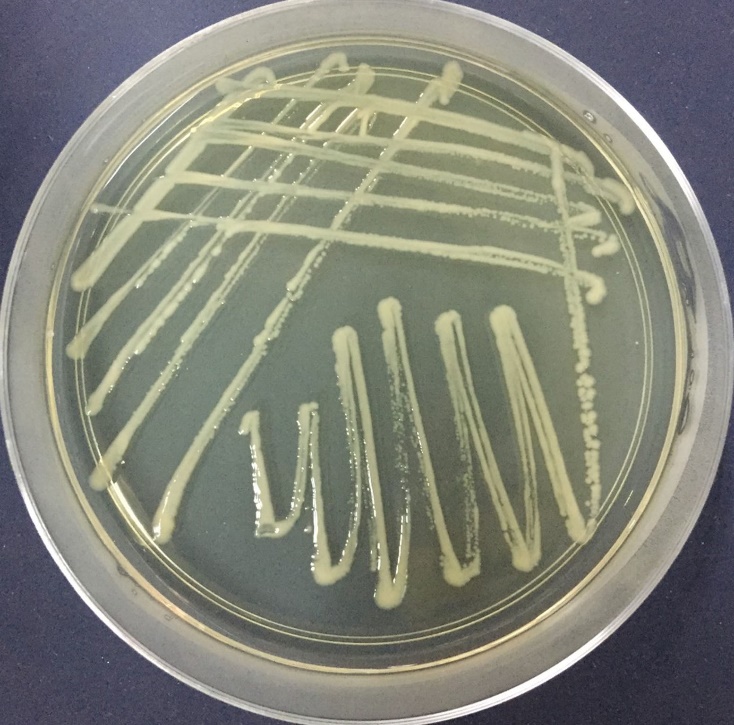


Fig. S1 *Cupriavidus necator* GX_5 colonies grown on LB agar

**Supplementary Table**

Table S1 The effect of initial Cd (II) concentration on adsorption capacity of *Cupriavidus necator* GX_5

| D Initial Cd^2+^ concentration (mg/L)  Adsorption capacity (mg/g)  Biosorbents | 5 | 10 | 20 | 50 | 100 | 200 |
| --- | --- | --- | --- | --- | --- | --- |
| Live | 3.03^h^±0.12 | 4.29^fgh^±0.03 | 5.16^efg^±0.26 | 7.52^cd^±0.54 | 11.25^b^±0.88 | 17.17^a^±1.62 |
| Dead | 3.93^gh^±0.03 | 5.42^ef^±0.14 | 6.46^de^±0.44 | 8.76^c^±0.43 | 12.02^b^±1.45 | 17.55^a^±1.70 |

Note: Values are means of three replications ± standard deviation;

Means with the same letter (superscripts) are not significantly different (*p* < 0.05), the same below.

Table S2 The effect of initial Cd (II) concentration on removal efficiency of *Cupriavidus necator* GX_5

| D Initial Cd^2+^ concentration (mg/L)  Removal efficiency (%)  Biosorbents | 5 | 10 | 20 | 50 | 100 | 200 |
| --- | --- | --- | --- | --- | --- | --- |
| Live | 60.51^b^±2.36 | 42.91^d^±0.31 | 25.78^f^±1.32 | 15.05^h^±1.08 | 11.25^i^±0.88 | 8.58^j^±0.81 |
| Dead | 78.53^a^±0.60 | 54.23^c^±1.44 | 32.32^e^±2.19 | 17.52^g^±0.86 | 12.02^i^±1.45 | 8.77^j^±0.85 |

Table S3 The effect of biosorbent dosage on adsorption capacity of *Cupriavidus necator* GX_5

| Dosage (g/L)  Adsorption capacity (mg/g)  Biosorbents | 0.2 | 0.5 | 1.0 | 2.0 | 4.0 |
| --- | --- | --- | --- | --- | --- |
| Live | 15.97^b^±1.30 | 9.34^de^±0.91 | 7.33^f^±0.64 | 6.44^fg^±0.06 | 4.39^h^±0.12 |
| Dead | 21.47^a^±2.16 | 13.15^c^±1.01 | 9.57^d^±0.48 | 7.87^ef^±0.09 | 5.44^gh^±0.19 |

Table S4 The effect of biosorbent dosage on removal efficiency of *Cupriavidus necator* GX_5

| D Dosage (g/L)  Removal efficiency (%)  Biosorbents | 0.2 | 0.5 | 1.0 | 2.0 | 4.0 |
| --- | --- | --- | --- | --- | --- |
| Live | 6.39^h^±0.52 | 9.34^g^±0.91 | 14.65^f^±1.27 | 25.77^d^±0.23 | 35.15^b^±0.98 |
| Dead | 8.59^g^±0.87 | 13.15^f^±1.01 | 19.13^e^±0.95 | 31.49^c^±0.38 | 43.49^a^±1.54 |
